# Association between serum phosphate levels and anemia in non-dialysis patients with chronic kidney disease: a retrospective cross-sectional study from the Fuji City CKD Network

**DOI:** 10.1186/s12882-023-03298-9

**Published:** 2023-08-22

**Authors:** Kazuhiko Kato, Akio Nakashima, Ichiro Ohkido, Kenji Kasai, Takashi Yokoo

**Affiliations:** 1 Division of Nephrology and Hypertension, Department of Internal Medicine, The Jikei University School of Medicine, 3-25-8 Nishi-Shimbashi Minato-Ku, Tokyo, 105-8461 Japan; 2 Department of Internal Medicine, Fuji City General Hospital, Takashima-Cho 50, Fuji-Shi, Shizuoka, Japan

**Keywords:** Anemia, Chronic kidney disease, Chronic kidney disease-mineral and bone disorder, Erythropoietin stimulating agent, Iron therapy, Serum phosphate

## Abstract

**Background:**

Patients with chronic kidney disease (CKD) present high mortality and morbidity rates despite the availability of various therapies. Although CKD-mineral and bone disorder (MBD) and renal anemia are important factors in patients with CKD, only few studies have analyzed the relationship between them. Therefore, this study aimed to evaluate the relationship between CKD-MBD and anemia in patients with CKD who did not receive erythropoiesis-stimulating agent or iron therapies.

**Methods:**

This retrospective cross-sectional study included patients with CKD aged ≥ 20 years with estimated glomerular filtration rate (eGFR) categories G2a to G5 who were referred to the Fuji City General Hospital between April 2018 and July 2019. The exclusion criterion was ongoing treatment for CKD-MBD and/or anemia.

**Results:**

The data of 300 patients with CKD were analyzed in this study. The median age of patients was 71 (range, 56.5–79) years. The median eGFR was 34 (range, 20–48) mL/min/1.73 m^2^, and the mean hemoglobin (Hb) level was 12.7 g/dL (standard deviation, 2.3), which decreased as the CKD stage increased. In a multivariate linear regression analysis of anemia-related factors, including age, renal function (eGFR), nutritional status, inflammation, and iron dynamics (serum iron level, total iron-binding capacity, ferritin levels), the serum phosphate levels were significantly associated with the Hb levels (coefficient [95% confidence interval], -0.73 [-1.1, -0.35]; *P* < 0.001). Subgroup analysis revealed a robust association between serum phosphate levels and Hb levels in the low-ferritin (coefficient [95% confidence interval], -0.94 [-1.53, -0.35]; *P* = 0.002) and advanced CKD groups (coefficient [95% confidence interval], -0.89 [-1.37, -0.41]; *P* < 0.001).

**Conclusions:**

We found an association between high serum phosphate levels and low Hb levels in patients with CKD not receiving treatment for anemia. These results underscore the possibility of a mechanistic overlap between CKD-MBD and anemia.

## Background

Anemia is a common complication of chronic kidney disease (CKD) [1] and an established risk factor for congestive heart failure and cardiovascular disease (CVD) [2, 3]. Previous studies have found that improvement in anemia inhibits CKD progression [4] and helps prevent CVD [5]. Anemia in patients with CKD, known as “renal anemia,” is caused by erythropoietin (EPO) deficiency, and current treatment strategies focus on this condition [1]. Renal anemia is caused by reduced EPO production owing to renal dysfunction. Recently, even mild renal dysfunction was reported to cause renal anemia [6]. However, causes for anemia in patients with CKD are multifactorial, including EPO deficiency, iron deficiency, inflammation, and vitamin D insufficiency [1, 7, 8].

Recently, several studies have found an association between CKD-mineral and bone disorder (CKD-MBD) and anemia [9]. A study of 2,089 patients with CKD stages 1–5 showed a significant correlation between high serum phosphate levels and anemia after performing a cross-sectional analysis [10], whereas another study of 2,930 patients with CKD stages 3–5 showed an association between high serum phosphate levels and anemia [11].

Fibroblast growth factor-23 (FGF23), a hormone that regulates bone metabolism, is assumed to be the key factor linking CKD-MBD and anemia. Interestingly, several clinical studies involving patients with CKD stages 3–4 [12] and 2–4 [13], along with patients undergoing hemodialysis [14], have demonstrated an association between FGF23 and anemia. However, these results should be interpreted with caution, as many evaluated patients were receiving iron and erythropoiesis-stimulating agent (ESA) therapies. To the best of our knowledge, no studies have reported on patients with CKD not treated with iron or ESA therapy; therefore, this study aimed to evaluate the relationship between CKD-MBD and anemia after excluding these factors.

In this study, we investigated the association between high serum phosphate levels and anemia in patients referred through the Fuji City CKD Network to examine whether an overlap between CKD-MBD and anemia exists. The Fuji City CKD Network was launched in 2013, based on collaboration between family physicians and nephrologists, to provide treatment for CKD. It aimed to reduce the initiation of dialysis and incidence of CVD in patients with CKD by early identification and appropriate treatment through referrals from their physicians. Many patients referred through this network had not been treated for CKD-MBD or renal anemia. Therefore, this group of patients was well suited for investigating the association between CKD-MBD and anemia, and we conducted this cross-sectional study to investigate the association between the serum phosphate and hemoglobin (Hb) levels.

## Methods

### Study population

This retrospective cross-sectional study included patients with CKD at the Fuji City General Hospital between April 2018 and July 2019. The inclusion criteria were as follows: patients aged ≥ 20 years with estimated glomerular filtration rate (eGFR) categories ranging from G2a to G5, based on the CKD guidelines [15], with or without abnormal urinary findings or renal imaging findings. The exclusion criterion was ongoing treatment for CKD-MBD and/or anemia.

### Clinical and laboratory data collection

We recorded the patients’ demographic data, comorbid conditions, and current medications at enrollment.

Different parameters, including the levels of blood creatinine (mg/dL), alkaline phosphatase (U/L), lactic acid dehydrogenase (U/L), total protein (g/dL), albumin (g/dL), sodium (mEq/L), potassium (mEq/L), urea nitrogen (mg/dL), calcium (mg/dL), phosphate (mg/dL), serum iron (µg/dL), total iron-binding capacity (µg/dL), ferritin (ng/mL), magnesium (mg/dL), and C-reactive protein (mg/dL), along with white blood cell count (× 10^3^/µL), red blood cell count (× 10^6^ /µL), Hb level (g/dL), hematocrit (%), mean corpuscular volume (fL), mean corpuscular hemoglobin concentration (g/dL), platelet count (× 103/µL), reticulocyte count (%), and hydrogen carbonate level (mmol/L) were tested using standard commercial assays. Spot urine samples were also obtained to determine the protein level. eGFR was calculated using the following formula for Japanese individuals:

eGFR (mL/min/1.73 m^2^) = 194 × (serum creatinine level [mg/dL])^−1.094^ × (age [years])^−0.287^ × (0.739 if female) [16].

### Statistical analyses

To determine the between-group differences, the chi-square test was used for categorical data and the Mann–Whitney U test and Student’s t-test for continuous data. Subsequently, the Hb levels for each CKD stage were shown using a box-and-whisker diagram. A multivariate linear regression analysis was performed to examine the independent association between the phosphate and Hb levels. In this analysis, we considered the following as confounding factors, based on previous clinical studies [10–14]: age, sex, comorbidities, renal dysfunction, poor nutrition, iron dynamics, inflammation, and hematopoietic potential. The variables related to renal dysfunction were eGFR, urea nitrogen level, and urinary protein level, and those related to poor nutrition were albumin level and body mass index. The variables related to iron dynamics included the serum iron level, total iron-binding capacity, and ferritin level, whereas those related to inflammation were the C-reactive protein level and white blood cell count. The variables related to hematopoietic potential included reticulocyte count. Further, calcium level was used as a variable related to phosphate level. We also confirmed that these variables were not multicollinear. Moreover, Spearman’s rank correlation coefficients and regression coefficients from univariate linear regression analysis were determined for the association between variables used in multivariate analysis and the Hb levels. Moreover, the association was evaluated using a three-knot restricted cubic spline analysis using similar explanatory variables. Next, a multivariate linear regression analysis with similar variables was performed as a sensitivity analysis, excluding patients with extremely high or low eGFR. The sensitivity analysis included patients with an eGFR < 60 mL/min/1.73 m^2^, which is the criterion for a low eGFR [15] and an eGFR ≥ 8 mL/min/1.73 m^2^, which is the lower limit for considering conservative treatment, according to the Japanese maintenance dialysis guidelines [17]. Finally, as subgroup analysis, a multivariate linear regression analysis with similar variables was performed for two group sets, i.e., the CKD stage 2–3 and CKD stage 4–5 groups, and for two groups divided by the median ferritin and phosphate levels. The low-ferritin and low-phosphate groups comprised patients with ferritin and phosphate levels < 136 ng/mL and < 3.4 mg/dL, respectively. In this study, all tests were two-sided, and a *P*-value < 0.05 was considered statistically significant. Complete case analysis was used where any data were missing. All statistical analyses were performed using the statistical software for social sciences, Stata Version 15.1 (College Station, TX, USA).

## Results

Between April 2018 and July 2019, 343 patients with CKD were referred to the Fuji City General Hospital through the Fuji City CKD Network. Of these, 326 patients were included in this study. Based on the exclusion criterion, we excluded 15 patients with medication related to CKD-MBD, such as vitamin D or phosphate binders, nine patients receiving treatment for anemia, including iron or ESA, and two patients receiving both treatments. Finally, a total of 300 patients with CKD were analyzed in the study.

**Fig. 1:**
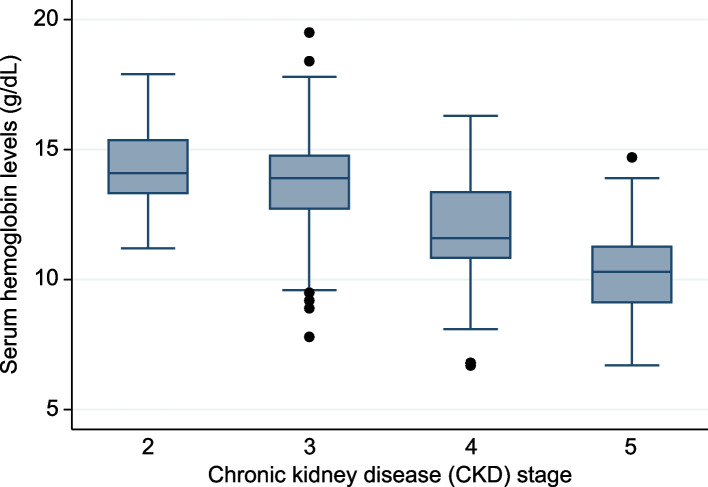
Hb levels classified by the CKD stage. Hb, hemoglobin; CKD, chronic kidney disease

Table 1 presents the patients’ background data based on the median phosphate level (3.4 mg/dL). The median age of patients was 71 (range, 56.5–79) years, including 92 (31%) patients with a history of diabetes. The median eGFR and calcium levels were 34 (range, 20–48) mL/min/1.73 m^2^ and 9.1 (range, 8.7–9.4) mg/dL, respectively. The median serum phosphate level was 3.3 (range, 3–3.8) mg/dL, and the mean Hb level was 12.7 g/dL (standard deviation, 2.3), which decreased as the CKD stage increased (Fig. 1).

**Table 1:** Characteristics of the 300 study patients based on their phosphate levels

Variables	Overall	Low-phosphate group (< 3.4 mg/dL)	High-phosphate group (≥ 3.4 mg/dL)	*P*-value
Sex, male [*n* (%)]	210 (70%)	126 (80%)	84 (60%)	< 0.001
Age (year)	71 (56.5–79)	69 (57–77)	72 (56–81)	0.065
Body mass index (kg/m^2^)	24.1 (21.3–26.5)	24.5 (22.2–26.4)	24.0 (21–26.5)	0.119
Smoking [*n* (%)]	124 (42%)	75 (48%)	49 (35%)	0.023
Cancer [*n* (%)]	35 (12%)	12 (9%)	23 (16%)	0.02
Diabetes [*n* (%)]	92 (31%)	34 (22%)	58 (41%)	< 0.001
Bone fracture [*n* (%)]	11 (4%)	5 (3%)	6 (4%)	0.24
Gastrointestinal bleeding [*n* (%)]	2(1%)	1 (1%)	1 (1%)	0.006
Creatinine (mg/dL)	1.54 (1.1–2.57)	1.29 (1.02–1.73)	2.21 (1.31–3.37)	< 0.001
eGFR (mL/min per 1.73 m^2^)	34 (20–48)	40 (28–54)	23 (14–38)	< 0.001
Urine protein (g/gCr)	0.7 (0.1–2.7)	0.3 (0.1–1.5)	1.6 (0.3–3.7)	< 0.001
Alkaline phosphatase (U/L)	243.5 (190–299)	239 (186–298)	246 (198–299)	0.477
Lactic acid dehydrogenase (U/L)	206 (177–241)	200 (176–225)	220 (179–261)	0.003
Total protein (g/dL)	7.2 (6.8–7.5)	7.2 (6.9–7.6)	7.1 (6.6–7.5)	0.013
Albumin (g/dL)	4 (3.6–4.3)	4.1 (3.7–4.3)	3.8 (3.4–4.2)	< 0.001
Sodium (mmol/L)	140 (138–141)	140 (139–142)	140 (138–141)	0.12
Potassium (mmol/L)	4.5 (4.2–4.9)	4.5 (4.1–4.8)	4.6 (4.2–5.0)	0.03
Urea nitrogen (mmol/L)	25 (18–37)	21 (16–27)	34 (21–47)	< 0.001
Uric acid (mg/dL)	6.3 ± 1.5	6 ± 1.4	6.6 ± 1.5	< 0.001
Calcium (mg/dL)	9.1 (8.7–9.4)	9.2 (8.8–9.4)	8 (8.3–9.3)	< 0.001
Phosphate (mg/dL)	3.3 (3–3.8)	3 (2.7–3.1)	3.8 (3.6–4.2)	< 0.001
Serum iron (μg/dL)	78 (62–103)	85 (66–111)	72 (58–96)	0.003
TIBC (μg/dL)	298 (264–337)	306 (273–339)	290 (252–328)	0.01
Ferritin (ng/mL)	135.6 (62.4–261.1)	141.2 (65–268.4)	130.5 (57.6–261.1)	0.676
Magnesium (mg/dL)	2.1 (1.9–2.3)	2 (1.9–2.2)	2.1 (1.9–2.3)	0.1
C-reactive protein (mg/dL)	0.09 (0.03–026)	0.07 (0.02–0.24)	0.11 (0.03–0.27)	0.334
White blood cell (× 1,000/μL)	6.1 (5.2–7.6)	6.1 (5.3–7.3)	6.3 (5.2–8)	0.311
Red blood cell (× 1,000,000/μL)	4.22 ± 0.79	4.46 ± 0.69	3.96 ± 0.81	< 0.001
Hemoglobin (g/dL)	12.7 ± 2.3	13.6 ± 2	11.8 ± 2.3	< 0.001
Hematocrit (%)	38.9 (34–42.6.)	41.4 (37.2–43.6)	35.2 (31.2–40.6)	< 0.001
Mean corpuscular volume (fL)	91.4 (88.2–93.4)	91.5 (88.8–94.5)	91.2 (87.9–94)	0.236
MCHC (g/dL)	33.2 (32.6–33.8)	33.3 (32.7–33.9)	33.2 (32.4–33.7)	0.063
Platelet (× 1,000/μL)	216 (183–259)	210 (175–248)	228 (187–265)	0.043
Reticulocytes (%)	1.15 (0.9–1.49)	1.1 (0.9–1.47)	1.21 (0.94–1.51)	0.255
Hydrogen carbonate (mmol/L)	23.2 (21.5–24.7)	23.9 (22.3–25.2)	22.4 (20.2–24.5)	< 0.001

*eGFR* estimated glomerular filtration rate, *TIBC* total iron-binding capacity, *MCHC* mean corpuscular hemoglobin concentration

Table 2 lists the correlation and regression coefficients for the association between the variables used in the multivariate analysis and the Hb levels. In addition to known anemia-related variables, the serum phosphate levels were also negatively correlated with the Hb levels.

**Table 2:** Correlation coefficients and univariate linear regression analysis of each variable to the hemoglobin levels

		Univariate linear regression analysis	
Variables	Correlation coefficient	Regression coefficient (95% CI)	*P-*value
Sex, female		-1.43 (-1.99, -0.88)	< 0.001
Age (year)	-0.46	-0.06 (-0.08, -0.05)	< 0.001
Body mass index	0.21	0.1 (0.04, 0.16)	0.001
Smoking		0.86 (0.34, 1.39)	0.001
Cancer		-1.38 (-2.18, -0.59)	0.001
Diabetes		-0.53 (-1.09, 0.04)	0.07
Bone fracture		-2.2 (-3.58, -0.82)	0.02
Gastrointestinal bleeding		0.93 (-2.31, 4.17)	0.573
eGFR (mL/min/1.73 m^2^)	0.62	0.07(0.05, 0.08)	< 0.001
Urine protein (g/gCr)	-0.37	-0.19 (-0.27, -0.12)	< 0.001
Albumin (g/dL)	0.5	1.45 (1.11, 1.84)	< 0.001
Calcium (mg/dL)	0.47	1.74 (1.35, 2.13)	< 0.001
Phosphate (mg/dL)	-0.44	-1.45 (-1.77, -1.13)	< 0.001
Serum iron (μg/dL)	0.38	0.03 (0.02, 0.03)	< 0.001
Total iron-binding capacity (μg/dL)	0.4	0.01 (0.01, 0.02)	< 0.001
Ferritin (ng/mL)	-0.12	-0.002 (-0.003, -0.001)	< 0.001
C-reactive protein (mg/dL)	-0.13	-0.15 (-0.3, -0.01)	0.034
White blood cell (× 1,000/μL)	-0.04	-0.1 (-0.21, 0.02)	0.093
Reticulocytes (%)	-0.15	-0.91 (-1.5, -0.32)	0.003

*CI* confidence interval, *eGFR* estimated glomerular filtration rate

Table 3 shows the results of the multivariate linear regression analysis with Hb as an objective variable. In the unadjusted model, serum phosphate levels showed a significant negative correlation with the Hb levels (coefficient [95% confidence interval (CI)], -1.45 [-1.77, -1.13]; *P* < 0.001). In Model 3 analysis with the addition of anemia-related factors, such as age, renal function, nutritional status, inflammation, and iron dynamics, the serum phosphate levels showed a significant negative correlation with the Hb levels (coefficient [95% CI], -0.73 [-1.1, -0.35]; *P* < 0.001).

**Table 3:** Multivariate linear regression analysis of phosphate levels to the hemoglobin levels (all patients, *n* = 300)

	Coefficient (95% CI)	*P*-value
Unadjusted	-1.45 (-1.77, -1.13)	< 0.001
Model 1^a^	-1.28 (-1.58, -0.98)	< 0.001
Model 2^b^	-0.72 (-1.11, -0.32)	< 0.001
Model 3^c^	-0.73 (-1.1, -0.35)	< 0.001

*CI* confidence interval

^a^Model 1: Sex, age, smoking, cancer, diabetes, bone fracture, and gastrointestinal bleeding were added

^b^Model 2: The estimated glomerular filtration rate, urea nitrogen level, urinary protein level, calcium level, albumin level, body mass index, and reticulocyte count were added

^c^Model 3: The serum iron level, total iron-binding capacity, ferritin level, C-reactive protein level, and white blood cell count were added

**Fig. 2:**
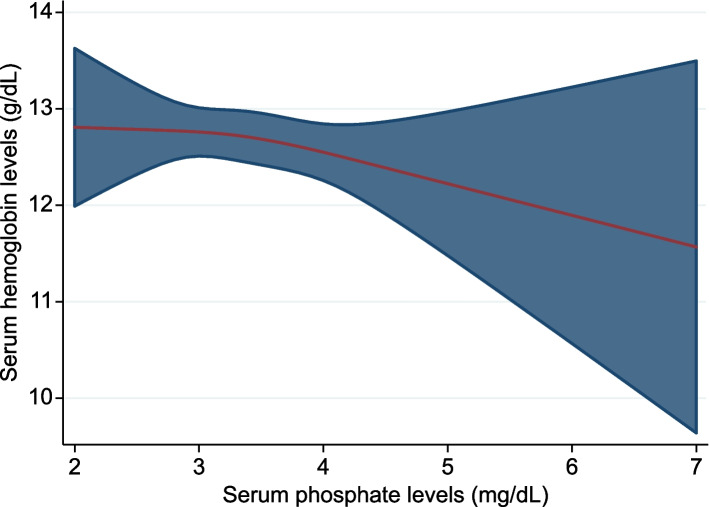
Cubic spline curve showing the association between the serum phosphate and Hb levels. Hb, hemoglobin

Figure 2 shows a restricted cubic spine curve showing the association between the serum phosphate and Hb levels. The Hb levels decreased as the phosphate levels increased.

Sensitivity analysis in patients with an eGFR < 60 mL/min/1.73 m^2^ and ≥ 8 mL/min/1.73 m^2^ revealed a significant negative correlation between the serum phosphate levels and Hb levels, similar to the results of the primary analysis (Table 4). The subgroup analysis (Tables 5, 6 and 7) revealed a robust negative correlation between high serum phosphate levels and the Hb levels in the low-ferritin and CKD stage 4–5 groups.

**Table 4:** Multivariate linear regression analysis of phosphate levels to the hemoglobin levels (patients with an eGFR < 60 mL/min/1.73 m^2^ and ≥ 8 mL/min/1.73 m^2^, *n* = 245)

	Coefficient (95% CI)	*P*-value
Unadjusted	-1.32 (-1.71, -0.93)	< 0.001
Model 1^a^	-1.13 (-1.49, -0.76)	< 0.001
Model 2^b^	-0.57 (-1.04, -0.11)	0.016
Model 3^c^	-0.69 (-1.12, -0.25)	0.002

*CI* confidence interval, *eGFR* estimated glomerular filtration rate

^a^Model 1: Sex, age, smoking, cancer, diabetes, bone fracture, and gastrointestinal bleeding were added

^b^Model 2: The estimated glomerular filtration rate, urea nitrogen level, urinary protein level, calcium level, albumin level, body mass index, and reticulocyte count were added

^c^Model 3: The serum iron level, total iron-binding capacity, ferritin level, C-reactive protein level, and white blood cell count were added

**Table 5:** Subgroup analysis of the multivariate linear regression of phosphate levels to the hemoglobin levels based on CKD stage

	Patients with CKD stages G2–3, *n* = 167		Patients with CKD stages G4–5, *n* = 133	
	Coefficient (95% CI)	*P*-value	Coefficient(95% CI)	*P-*value
Unadjusted	-0.62 (-1.17, -0.07)	0.028	-1.02 (-1.4, -0.64)	< 0.001
Model 1^a^	-0.26 (-0.75, 0.23)	0.29	-1.11 (-1.5, -0.73)	< 0.001
Model 2^b^	0.06 (-0.58, 0.71)	0.85	-0.92 (-1.46, -0.37)	0.001
Model 3^c^	-0.23 (-0.87, 0.41)	0.483	-0.89 (-1.37, -0.41)	< 0.001

*CI* confidence interval, *CKD* chronic kidney disease

^a^Model 1: Sex, age, smoking, cancer, diabetes, bone fracture, and gastrointestinal bleeding were added

^b^Model 2: The estimated glomerular filtration rate, urea nitrogen level, urinary protein level, calcium level, albumin level, body mass index, and reticulocyte count were added

^c^Model 3: The serum iron level, total iron-binding capacity, ferritin level, C-reactive protein level, and white blood cell count were added

**Table 6:** Subgroup analysis of the multivariate linear regression of phosphate levels to the hemoglobin levels based on the ferritin levels

	Low-ferritin group, *n* = 141		High-ferritin group, *n* = 140	
	Coefficient (95% CI)	*P*-value	Coefficient (95% CI)	*P*-value
Unadjusted	-1.32 (-1.76, -0.88)	< 0.001	-1.56 (-2.04, -1.08)	< 0.001
Model 1^a^	-1.14 (-1.56, -0.71)	< 0.001	-1.27 (-1.72, -0.83)	< 0.001
Model 2^b^	-0.99 (-1.55, -0.42)	0.001	-0.61 (-1.2, -0.02)	0.044
Model 3^c^	-0.94 (-1.53, -0.35)	0.002	-0.56 (-1.11, -0.01)	0.047

*CI* confidence interval

^a^Model 1: Sex, age, smoking, cancer, diabetes, bone fracture, and gastrointestinal bleeding were added

^b^Model 2: The estimated glomerular filtration rate, urea nitrogen level, urinary protein level, calcium level, albumin level, body mass index, and reticulocyte count were added

^c^Model 3: The serum iron level, total iron-binding capacity, ferritin level, C-reactive protein level, and white blood cell count were added

**Table 7:** Subgroup analysis of the multivariate linear regression of phosphate levels to the hemoglobin levels based on phosphate levels

	Low-phosphate group, *n* = 157		High-phosphate group, *n* = 141	
	Coefficient (95% CI)	*P*-value	Coefficient(95% CI)	*P*-value
Unadjusted	-0.49 (-1.5, 0.52)	0.341	-1.51 (-2.07 -0.95)	< 0.001
Model 1^a^	0.21 (-0.68, 1.09)	0.644	-1.73 (-2.24, -1.22)	< 0.001
Model 2^b^	-0.07 (-1.23, 1.09)	0.903	-0.72 (-1.42, -0.02)	0.045
Model 3^c^	-0.05 (-1.2, 1.1)	0.929	-0.79 (-1.45, -0.13)	0.019

*CI* confidence interval

^a^Model 1: Sex, age, smoking, cancer, diabetes, bone fracture, and gastrointestinal bleeding were added

^b^Model 2: The estimated glomerular filtration rate, urea nitrogen level, urinary protein level, calcium level, albumin level, body mass index, and reticulocyte count were added

^c^Model 3: The serum iron level, total iron-binding capacity, ferritin level, C-reactive protein level, and white blood cell count were added

## Discussion

In this study, we performed a cross-sectional analysis in a group of patients with CKD stages 2–5 who were not receiving treatment for anemia and/or CKD-MBD. We found a significant correlation between high serum phosphate levels and anemia, even after adjusting the effects of factors already known to be involved in anemia, including renal function, nutritional status, and iron metabolism. This result suggests a factor-mediated overlap between CKD-MBD and anemia. The strength of this study was that it presented this association in patients who had not undergone ESA and iron therapy, considering the effect of iron dynamics.

Patients with exceedingly elevated eGFR or an eGFR low enough to necessitate dialysis might not be suitable for this analysis, which aimed to examine the association between serum phosphate levels and anemia in stable non-dialysis CKD patients. Consequently, a sensitivity analysis was performed to exclude these individuals, revealing a significant correlation and supporting the findings of the primary analysis.

Previous studies have reported that FGF23 is a factor possibly associated with the overlap between CKD-MBD and anemia. It is a polypeptide hormone secreted by osteoclasts and other organs that suppresses the renal reabsorption and intestinal absorption of phosphate [18] and suppresses renal vitamin D synthesis [19], thereby decreasing blood phosphate level. FGF23 is thought to be a sensitive marker for the onset and progression of CKD, as its level is elevated earlier than is the serum phosphate level in response to phosphate load [20]. It is also an independent risk factor for CKD development [21]. Recently, elevated FGF23 levels were found to be associated with adverse outcomes, such as vascular calcification [22], left ventricular hypertrophy [23], cardiovascular events [24], and increased mortality [25, 26].

A previous study showed that various physiological mechanisms are involved in FGF23 activity in anemia [27]. First, FGF23 inhibits proerythroblasts from maturing into erythrocytes. Second, it reduces EPO secretion from the kidney, thereby decreasing the differentiation of erythroid progenitors [28]. Third, it promotes excessive hepcidin expression, which affects the erythrocytes in the G2/M phase of their cell cycle and enhances erythrocyte apoptosis.

Clinical studies have shown an association between FGF23 and anemia. A cross-sectional study of 53 patients with CKD stages 3 and 4 showed a negative correlation between the serum FGF23 level and Hb level [12]. The findings of a prospective cohort study of 3,869 patients with non-dialysis CKD indicated significant associations between elevated FGF23 levels and anemia [13]. A retrospective cohort study of 2,089 patients with non-dialysis CKD published in 2018 reported that high FGF23 levels were associated with anemia in a cross-sectional analysis; moreover, it showed an association between high serum FGF23 levels and an increased risk of developing anemia in a longitudinal analysis [10]. In a prospective cohort study of 1,044 patients undergoing hemodialysis, higher and lower FGF23 levels were associated with higher odds of ESA-hyporesponsiveness, although the association did not reach statistical significance [14]. These study findings suggest a physiological overlap between CKD-MBD and anemia through factors, such as FGF23. However, these studies included patients who were already being treated for anemia and did not show a pure association that was unaffected by treatment. As FGF23 is associated with iron metabolism and inflammation [29], the administration of ESA [30] and iron preparations [31] can affect FGF23 levels; therefore, we sought to evaluate the relationship after excluding these effects.

In this study, we performed a multivariate linear regression analysis, including iron kinetics and inflammation in a group of patients without any treatment effect on anemia and CKD-MBD, to eliminate the impact of treatment as far as possible. Our results showed a significant correlation between high serum phosphate levels and anemia, even after adjusting the effects of factors already known to be involved in anemia, such as renal function, nutritional status, and iron metabolism. This finding underscores the possibility of mechanistic overlap between CKD-MBD and anemia.

The subgroup analysis showed a robust correlation between high serum phosphate levels and anemia in the low-ferritin and CKD stage 4–5 groups, indicating a possible interaction of association between high serum phosphate levels and anemia in these groups. Nevertheless, both eGFR and ferritin level were not significant in the test of interaction (*P* = 0.336 and *P* = 0.398, respectively). However, the sample size in this study was not adequately powerful for conclusive interaction test results; thus, these interactions cannot be ruled out. Concerning the physiological mechanism of the interaction between phosphate and ferritin, iron deficiency may strengthen the association between CKD-MBD and anemia through FGF23, whose level is elevated during iron deficiency [32]. Unfortunately, no subgroup analysis of iron kinetics was conducted in previous studies, making it difficult to compare the results. Nevertheless, our findings suggest that iron-deficient patients with CKD may benefit from appropriate iron supplementation and strict management of CKD-MBD to prevent the development and progression of renal anemia. In contrast, the possibility of an interaction between phosphate and renal function may indicate that it is difficult to generalize the present results to the entire non-dialysis CKD population.

The association between phosphate and anemia shown in this study may be mediated by factors other than FGF23. While anemia is a risk factor for the development of CVD, CVD may also, in turn, increase the risk of anemia via elevated levels of inflammatory cytokines and other factors [33]. Conversely, phosphate promotes vascular calcification and increases the risk of CVD. Therefore, the possibility of phosphate levels being associated with anemia through increased arteriosclerosis and cardiovascular risk cannot be ruled out. In addition, parathyroid hormone (PTH) inhibits erythropoiesis and promotes osmotic fragility of erythrocytes through calcium-ATPase stimulation [34]; thus, it is conceivable that high-phosphate levels may have promoted PTH secretion and affected anemia. However, we were unable to obtain PTH data and examine this effect in this study.

There were several other limitations to our study. First, because of the observational nature of the study design, causality cannot be inferred, and there may be unmeasured confounders. Moreover, this study was a cross-sectional analysis; therefore, we could not examine changes over time. Second, we have not examined factors that link CKD-MBD and anemia, such as FGF23 and hepcidin. Third, the present data are limited to a single center and are not representative of all CKD populations. Fourth, we did not measure nutritional status indicators other than albumin. Finally, vitamin D deficiency affects renal anemia; however, we could not obtain data on 25-hydroxy vitamin D and 1–25-dihydroxy vitamin D levels. Thus, the effect of vitamin D deficiency on the association between CKD-MBD and anemia could not be considered in this study.

## Conclusions

A significant correlation was noted between high serum phosphate levels and anemia in patients with CKD who had not received treatment for anemia and CKD-MBD, even after excluding the effects of factors already known to be involved in anemia, such as renal function, nutritional status, and iron metabolism. This result underscores the possibility of a mechanistic overlap between CKD-MBD and anemia. Further prospective studies are required to elucidate the factors underlying this overlap in the same patient group.

## Data Availability

The datasets used during the current study are available from the corresponding author on reasonable request.

## Abbreviations

CI: Confidence interval
CKD: Chronic kidney disease
CVD: Cardiovascular disease
eGFR: Estimated glomerular filtration rate
EPO: Erythropoietin
ESA: Erythropoiesis-stimulating agents
FGF23: Fibroblast growth factor-23
Hb: Hemoglobin
MBD: Mineral and bone disorder
PTH: Parathyroid hormone
